# Religious activity and religious delusions in schizophrenia/schizoaffective disorder

**DOI:** 10.1192/j.eurpsy.2025.2193

**Published:** 2025-08-26

**Authors:** N. Ait Bensaid, M. Sabir, F. El Omari

**Affiliations:** 1 arrazi psychiatric hospital, Sale, Morocco; 2 HAS, arrazi psychiatric hospital, Sale, Morocco

## Abstract

**Introduction:**

Religious delusions are a common symptom in patients with schizophrenia. They can be more difficult to treat than other delusions because they are usually held with more conviction [1], which makes them of great clinical relevance. Religious delusions occur in between one-fifth and two-thirds of patients with delusions [2].

Cross-cultural differences may provide a partial explanation, but it seems likely that individual socio-demographic variables, particularly the extent of personal religiosity, as well as genetic factors, may also play a role.

**Objectives:**

To assess the relationship between sociodemographic characteristics and religious activity in patients with schizophrenia/schizoaffective disorder with religious delusions followed and hospitalised at the Arrazi psychiatric hospital in Salé.

**Methods:**

This was a descriptive cross-sectional study using a questionnaire including socio-demographic criteria, clinical criteria and questions about religious activity by grouping patients into 5 categories: (1 = no religious affiliation, 2 = religious affiliation, but not actively religious, 3 = religious affiliation, somewhat active activity, 4 = religious affiliation, moderately active activity, 5 = religious affiliation, very active activity) to assess the relationship between sociodemographic characteristics and religious activity in patients with schizophrenia/schizoaffective disorder with religious delusions followed and hospitalised at the Arrazi psychiatric hospital in Salé.

The inclusion criteria were as follows: both sexes with a diagnosis of schizophrenia/schizoaffective disorder according to DSM 5 criteria and having a mystico-religious delusion.

The exclusion criterion was severe intellectual disability.

**Results:**

A total of 109 patients were collected.

Approximately 85% were male. Most had an average socio-economic status. 67% lived with their families and 15 were homeless. About 89% were unemployed.

About 78% of the patients were hospitalised and most had poor compliance. All patients had mystico-religious delusions, most thought they were prophets or angels, 10 believed they were God. Fourteen patients thought they were Al Mehdi AL montadar, 2 said they were the Holy Spirit. 22 had a delusion of possession.

77% had Islam as their religion, 12% Christianity and 11% no religious affiliation. About half of the patients had a religious affiliation but were not actively religious. Very active religious subjects were 3 times more likely to suffer from religious delusions than subjects with no religious affiliation.

**Conclusions:**

Our data suggest that a high level of personal religious activity appears to be one of the risk factors for the onset of religious delusions. A high level of religiosity appears to increase the risk of developing religious delusions.

Further research is needed to examine the relationship between religiosity and religious delusions.

**Disclosure of Interest:**

None Declared

